# A Concise Review of Current In Vitro Chemical and Cell-Based Antioxidant Assay Methods

**DOI:** 10.3390/molecules26164865

**Published:** 2021-08-11

**Authors:** Ifeanyi D. Nwachukwu, Roghayeh Amini Sarteshnizi, Chibuike C. Udenigwe, Rotimi E. Aluko

**Affiliations:** 1 School of Public Health, Loma Linda University, Loma Linda, CA 92350, USA; inwachukwu@llu.edu; 2 School of Nutrition Sciences, Faculty of Health Sciences, University of Ottawa, Ottawa, ON K1H 8M5, Canada; roghayehamini66@gmail.com (R.A.S.); cudenigw@uottawa.ca (C.C.U.); 3 Department of Food Science and Technology, Faculty of Agriculture, Tarbiat Modares University, Tehran 14115-111, Iran; 4 Department of Chemistry and Biomolecular Sciences, Faculty of Science, University of Ottawa, Ottawa, ON K1N 6N5, Canada; 5 Department of Food and Human Nutritional Sciences, University of Manitoba, Winnipeg, MB R3T 2N2, Canada; 6 Richardson Centre for Functional Foods and Nutraceuticals, University of Manitoba, Winnipeg, MB R3T 2N2, Canada

**Keywords:** reactive oxygen species, antioxidant assays, free radicals, lipid peroxidation, radical scavenging, cell culture

## Abstract

Antioxidants remain interesting molecules of choice for suppression of the toxic effects of free radicals in foods and human systems. The current practice involves the use of mainly synthetic molecules as potent antioxidant agents. However, due to the potential negative impact on human health, there is an intensive effort within the research community to develop natural alternatives with similar antioxidant efficacy but without the negative side effects of synthetic molecules. Still, the successful development of new molecules depends on the use of reliable chemical or cell culture assays to screen antioxidant properties. Chemical antioxidant assays include the determination of scavenging ability against free radicals such as DPPH, superoxide anion radicals, hydroxyl radicals, hydrogen peroxide, and nitric oxide. Other antioxidant tests include the ability of compounds to bind and sequester prooxidant metal cations, reduce ferric iron, and attenuate the rate of lipid oxidation. Ex vivo tests utilize cell cultures to confirm entry of the molecules into cells and the ability to quench synthetic intracellular free radicals or to stimulate the increased biosynthesis of endogenous antioxidants. In order to assist researchers in their choice of antioxidant evaluation methods, this review presents background scientific information on some of the most commonly used antioxidant assays with a comparative discussion of the relevance of published literature data to food science and human nutrition applications.

## 1. Introduction

Reactive oxygen species (ROS) are formed endogenously in the human body as part of everyday physiological processes, by exogenous physiochemical processes or as a result of pathological conditions. Often denoted as the villain in antioxidant-related discussions, ROS are actually useful and essential components of the body’s metabolic machinery since they contribute to modulating critical physiological processes such as cell cycle progression, intracellular signal transduction, and gene expression [1,2]. At normal physiological levels, ROS such as hydroxyl radicals and hydrogen peroxide are harmlessly neutralized in biological systems using well-developed endogenous antioxidant defense systems [3,4], including enzymatic (glutathione peroxidase, catalase, and superoxide dismutase) and non-enzymatic (glutathione, tocopherol, ascorbic acid, melatonin) antioxidants [4,5,6]. For proper physiological functions, it is important to maintain a normal balance between the ROS (including free radicals, i.e., molecules containing an unpaired electron in their external molecular orbitals) produced in the body and the body’s antioxidant molecules. This is because a sustained overproduction of ROS could overwhelm the body’s natural defense system [4,7], resulting in oxidative stress and possibly oxidative damage to important cellular and extracellular macromolecules (i.e., proteins, lipids, and nucleic acids) with the potential for adverse consequences such as atherosclerosis, inflammation, cancer, arthritis, Alzheimer’s, Parkinson’s, neoplasia, and aging [7,8,9]. 

Given the widely reported toxicity of synthetic antioxidants such as butylated hydroxytoluene and butylated hydroxyanisole as well as consumers’ demand for ‘clean label’ all-natural antioxidants [6,10], investigators have worked to direct attention and efforts toward using food-derived exogenous antioxidants to complement and enhance the human endogenous antioxidant defense system [8]. It has been suggested that protein-derived antioxidative peptides could serve as ingredients in foods in order to help reduce the potential for oxidative stress-related chronic disease conditions [8,11] and be included as additives in order to minimize the oxidation of food products [12]. Given that lipid oxidation is well recognized as the major deterioration process that impairs the nutritional and sensory quality of foods [10], particularly lipid-based food products, the application of antioxidative food ingredients in this manner could help prevent oxidative rancidity and preserve the nutritional, organoleptic, and shelf life of foods [11,12,13].

The last few decades have seen a significant rise in attention and research efforts toward the field of free radical and antioxidant chemistry given the critical role of antioxidants in food, medicine, and health [6,14]. For instance, antioxidants are critical to certain food preservation techniques where they are used to inhibit oxidation processes, while vitamins, polyphenols, and carotenoids are all well-known antioxidants of dietary origin [9,10]. Thus, antioxidants are important contributors to food security, safe and adequate nutrition, as well as health promotion. Interest in determining and quantifying antioxidants in foods and biological samples has continued to grow due to the need to identify and develop effective molecules, which could serve as a shield against oxidative stress-related chronic disease conditions [6,8,10,14,15,16]. However, questions have perennially surrounded the reliability, accuracy, validity, and usefulness of existing antioxidant assays, with some of the most persistent questions being the low applicability and poor correlation between in vitro and in vivo assay results [10,14,17]. Therefore, this review was undertaken with the objective to provide a concise discussion of current antioxidant assays, their limitations, suggested improvements, and recommendations for modifications that could lead to improved accuracy and reliability.

## 2. Principles of Chemical Antioxidant Assays 

In vitro chemical antioxidant assays can generally be classified into two: those involving hydrogen atom transfer (HAT) and those based on electron transfer (ET) [18]. While most HAT reactions such as lipid peroxidation (including lipoprotein oxidation) and oxygen radical absorbance capacity (ORAC) measure the capacity of an antioxidant to inactivate a free radical through the release of a hydrogen atom, assays based on single electron transfer such as ferric iron reducing antioxidant power (FRAP), 2,2-diphenyl-1-picrylhydrazyl (DPPH), and 2,2′-azinobis[3-ethylbenzothiazoline-6-sulfonic acid] (ABTS) measure the release or transfer of an electron to a free radical, thus converting it into an anion [18,19]. A number of technical and conceptual impediments that preclude the use and impair the validity of in vitro antioxidant assays have been discussed [17]. These limitations include the unsuitability of the chemistry and molecular targets of most in vitro assays to the in vivo environment, the inadequacy of commonly used antioxidant assays in measuring the radical reactions in lipids, the ambiguity of the chemistry of the assays with regard to quantitative analyses, and the near absence of standardization in the experimental procedures of many currently used antioxidant assays [17]. With respect to the poor correlation between the chemistry and molecular targets of in vitro assays and that of the in vivo environment, for instance, it has been noted that the direct scavenging of the extremely reactive and short-lived hydroxyl radical (^•^OH) by dietary antioxidants in vivo is unrealistic and of diminished physiological relevance. This is because the intracellular concentration of dietary antioxidants is negligible and thus insufficient to scavenge this most powerful biological oxidizing intermediate [20], which has the capacity to hydroxylate biological macromolecules such as proteins, nucleic acids, and lipids [18]. In addition, it is recognized that careful consideration must be given to the type of solvent used in antioxidant assays in order to avoid false positive results [15], since the type of solvent used could significantly affect test results [10]. For instance, in a study examining the effect of various solvent types viz methanol, isopropanol, chloroform, acetone, hexane, and ethyl acetate on the amount of unreacted DPPH radical, the highest concentration of DPPH radical remaining after a reaction time of 60 min was observed for ethyl acetate and the lowest was observed for chloroform [21]. While the DPPH reagent is notable for its affinity toward hydrophobic solvents in contradistinction to its antipathy for hydrophilic ones, the ABTS reagent can effectively dissolve in both lipophilic and hydrophilic solvents [22]. The sub-sections that follow provide a concise discussion of the various bases of chemical antioxidant assays such as scavenging of free radicals, trapping of reactive oxygen species, reduction of ferric iron to the more stable Fe^2+^ form, prooxidant chelation of metal ions, and inhibition of lipid peroxidation.

### 2.1. Free Radical (Synthetic Dpph) Scavenging

In living cells, a natural antioxidant defense system consisting of endogenous enzymatic antioxidants exists to counteract the actions of ROS such as superoxide anion and hydrogen peroxide, while a variety of non-enzymatic antioxidants such as ascorbic acid and α-tocopherols are tasked with the responsibility of scavenging free radicals and oxidants such as peroxynitrite, hydroxyl radical, singlet oxygen, and peroxyl radicals [10,18,23]. Since they are rapid, simple, relatively straightforward, and largely inexpensive, a number of antioxidant assays designed to measure the capacity of antioxidants to scavenge free radicals are widely used in food and biological science research to evaluate the free radical/oxidant-scavenging property of biological and food samples [15,18,23].

**Figure 1:**
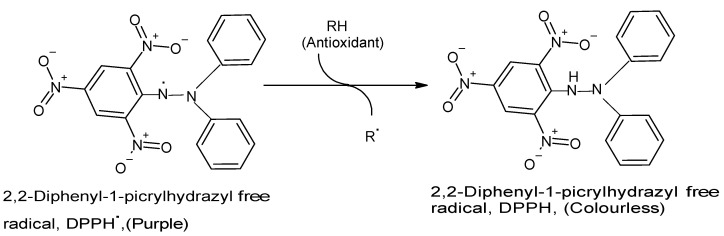
Reaction between DPPH radical and an antioxidant to yield the colorless DPPH. The reaction of DPPH radical with other radicals, hydrogen atoms, or electrons results in the loss of color at 515 nm. (Chemical structures produced with ACD/ChemSketch Freeware Version 2021.1.0 C25E41).

The DPPH assay has become one of the most commonly used in vitro chemical antioxidant tests mainly because it is highly sensitive, technically simple, rapid, accurate, reproducible, reliable, and does not require any special sample pre-treatment [10,15,23,24,25]. The assay is usually performed by combining a methanolic DPPH solution (25 mg/L) with the test sample solution and monitoring the absorbance of the mixture at 515–517 nm using a spectrophotometer for 30 min or until the absorbance is stable [15,18]. A strong (purple) absorption maximum is shown by the DPPH organic nitrogen radical at 517 nm, and the antioxidant capacity of the test sample is directly proportional to the disappearance of color, i.e., reduced absorbance [15,23]. Upon reduction of the DPPH radical by and following hydrogen atom abstraction from the antioxidant, the solution loses its color and fades from purple to pale yellow [15,23] (Figure 1). Then, absorbance (A_s_) of the reaction containing the antioxidant molecule is subtracted from absorbance (A_b_) of the reaction without the sample (blank) and expressed as percentage loss. When the reaction is performed at different sample (antioxidant) concentrations, a plot of inhibition rate versus concentration allows for calculation of the EC_50_ (effective concentration of the antioxidant needed to reduce the amount of DPPH radicals by half) [17].

In contrast to most other free radicals, the DPPH molecule does not undergo dimerization because the spare electron is delocalized over the entire molecule, resulting in the formation of a deep violet color in aqueous, methanolic, and ethanolic solutions in which the radical has been shown to rarely disintegrate [25]. Since DPPH is hydrophobic, assays involving the free radical must be performed in organic solvents [17]. The characteristics of the selected solvent and pH can influence DPPH scavenging activity, thus highlighting the importance of carefully considering solvent property in order to avoid false positive results [15]. It was reported that in the reaction between DPPH and phenols, the rate-determining step is the very rapid transfer of electron from the phenoxide anions to DPPH, while the hydrogen atom transfer from the neutral phenol to DPPH represents a marginal reaction step, since it occurs at a much slower rate in strong hydrogen bond-accepting solvents such as alcohol and methyl alcohol [26]. In a study of crude palm oil-derived carotenoids, the finding that α- and β-carotenes were better DPPH radical scavengers than metal chelators was suggested to be probably due to the presence of unsaturated groups in the tetraterpenoids [27]. Limitations of the DPPH assay include that it misses critical data present in reaction curves, since it does not measure reaction rates as well as the interaction of DPPH with dissolved oxygen, which could be an issue for compounds that autoxidize such as certain phenols and ascorbate [17]. In addition, the steric inaccessibility of the DPPH radical site, which impairs its interaction with samples such as fruits and vegetables extracts that typically contain a mixture of antioxidants also limits the reliability of the test [17]. For instance, samples containing eugenol and similar phenols with the o-methoxyphenol structure are known to give falsely low antioxidant capacity readings [18]. Results from the use of DPPH as a colorimetric probe for the detection of free radical scavengers are often reported as EC_50_ or as TEC_50_, which is defined as the time required to reach steady state with EC_50_ [10]. However, the usefulness of interpreting DPPH assay data in this manner has been called into question, since EC_50_ is time-dependent, and the effect of time is not uniform for all compounds, and also because EC_50_ is a concentration, not a kinetic parameter, and therefore cannot accurately be used to denote the antioxidant or antiradical capacity of a compound [28]. Moreover, the DPPH radical is a synthetic compound that is not present in plant and animal tissues; therefore, the data obtained may not reflect the true radical scavenging ability of a molecule within the human body or as food preservatives. Hence, extrapolation of the DPPH radical scavenging data to the antioxidant capacity of a molecule in real life or food product situations must be done with caution.

### 2.2. Ros Trapping (Hydrogen Peroxide, Nitric Oxide, Also Superoxide and Hydroxyl)

Of the six major ROS (superoxide anion, hydrogen peroxide, peroxyl radicals, hydroxyl radical, singlet oxygen, and peroxynitrite) known to cause oxidative damage in the human body, two endogenous antioxidant enzymes, namely superoxide dismutase and catalase, are known to neutralize superoxide anion and hydrogen peroxide, respectively [18,26]. In contrast, non-enzymatic antioxidants such as phytochemicals, ascorbic acid, and alpha-tocopherol are saddled with the responsibility of scavenging the other four oxidants and free radicals [18,29]. Various in vitro ROS trapping assays exist for evaluating the oxidant scavenging capacity of biological samples. For instance, the hydroxyl radical scavenging activity of a range of biological samples such as chicken skin enzymatic protein hydrolysates [30] and peach fruit extracts [31] has been evaluated using a method proposed by de Avellar et al. [32]. This method is based on the production of hydroxyl radicals in the Fenton reaction following the combination of hydrogen peroxide with 1,10-phenanthroline and ferrous ammonium sulfate. Although hydrogen peroxide is chemically unreactive at low concentrations, under physiological conditions, its oxidation power can be observed in combination with ferrous ion in the Fenton reaction [18]. With regard to the unsuitability of the chemistry and molecular targets of in vitro assays to in vivo reactions, for instance, it has been noted that the Fe^2+^/H_2_O_2_ mixture used in scavenging assays has a certain drawback, since many antioxidants are also metal chelators [18]. Thus, mixing the antioxidant sample with Fe^2+^ could alter the activity of the ferrous ion by chelation, thus making it impossible to determine if the antioxidant is an effective hydroxyl radical scavenger or just a good metal chelator [18].

### 2.3. Ferric Reducing Antioxidant Power (FRAP)

**Figure 2:**
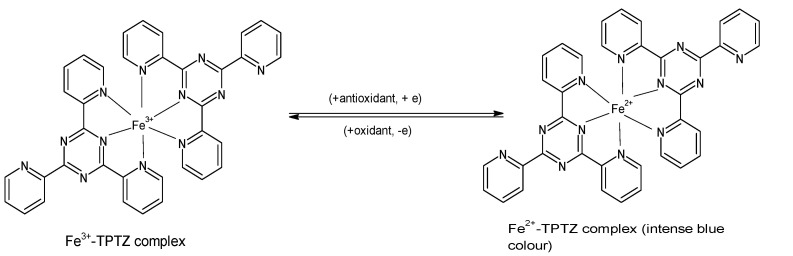
Reaction of ferric tripyridyl-S-triazine complex with antioxidant to yield the intensely blue ferrous form of the complex at an absorbance maxima of 593 nm. (Chemical structures produced with ACD/ChemSketch Freeware Version 2021.1.0 C25E41).

The FRAP assay evaluates the ability of antioxidants to reduce ferric iron in the form of Fe^3+^-2,4,6-tripyridyl-S-triazine (TPTZ) complex to the more stable, divalent Fe^2+^ ion at low pH [8,33]. Initially developed to determine the concentration of ascorbic acid in plasma [34], the assay measures the change in absorbance using a spectrophotometer, and it is performed by incubating 300 µL of freshly prepared “FRAP reagent” (25 mL acetate buffer, 2.5 mL FeCl_3_**·**H_2_O (20 mM), and 2.5 mL of 10 mM TPTZ in 40 mM HCl) with a reagent blank at 37 °C for 30 min, and taking a reading at 593 nm. Subsequently, the test sample (10 µL) and water (30 µL) are added to the reaction mixture, and absorbance readings are taken after 0.5 s and thereafter, every 15 s for 4 min [15,18]. The reduction to Fe^2+^ that yields a violet-blue color (Figure 2) provides a quick, reproducible result and has been used in many studies for determining the antioxidant capacity of various foods including vegetables, cereals, fruits, beans, and essential oils [23,35,36,37,38,39]. Certain polyphenols such as ascorbic acid, quercetin, ferulic acid, caffeic acid, and tannic acid have been reported to show increasing absorbance (A_593_) beyond the standard assay reaction time of 4 min, thus the higher FRAP values of such compounds [40].

In food protein-derived bioactive peptides, certain properties such as a terminal methionine residue, the presence of sulfur-containing amino acids, and amino acid hydrophobicity have been reported to enhance FRAP. In contrast, the presence of lysine residues and a high content of cationic amino acids in protein hydrolysates is thought to impair their FRAP potential [3,8,41,42]. In a study that investigated various antioxidant activities of 13 apple cultivars, the highest FRAP values were recorded for the apple peel extracts, which contain higher phenolics and flavonoids, and lower ascorbic acid levels compared to the samples from the apple cortex [35]. The ferric reducing capacity (FRC) assay, which is similar to the FRAP assay in many aspects including in lacking the capacity to measure thiol groups, but which differs from the latter in replacing TPTZ with 1,10-phenanthroline (since phenanthroline forms a Fe^3+^-[Phen]_3_ complex that is reduced to an orange-red Fe^2+^-(Phen) complex), has been used as an alternative to FRAP due to its simplicity and speed [9]. In a study comparing the performance of both assays in measuring the total antioxidant capacity (TAC) of serum samples, serum TAC values ranged from 172 to 418 μmol/L of vitamin C equivalents for FRAP and from 264 to 610 for FRC, with a Spearman rank order correlation result (rs = 0.75, *p* = 0.01) suggesting a strong positive correlation between the TAC of the serum samples measured by both assay methods [9]. Another study compared the antioxidant activities of medicinal plant infusions using a modified FRC assay (the highly ferrous-stabilizing ligand ferrozine) and the conventional FRAP test [43]. It was found that the FRC was superior to FRAP with respect to faster kinetics, enhanced sensitivity, and absence of free Fe (II), which has been shown to lead to Fenton-type oxidations in reaction products. In the modified FRC assay, ferric ion, in the presence of ferrozine, is reduced to the magenta colored Fe^2+^-ferrozine complex with absorption maxima at 562 nm [43]. A more recent modification of the FRAP assay in which the spectrophotometric quantification of the Prussian blue end product is used to determine antioxidant reducing power involves the use of potassium ferricyanide [10]. In this iteration of the FRAP assay, the antioxidant sample either reduces the ferric ion in the solution to ferrous ion, which then complexes with the ferricyanide to yield Prussian blue or reduces the ferricyanide to ferrocyanide that interacts with the free ferric ion in the solution to form Prussian blue [10,44].

Certain limitations impair the validity, efficiency, usefulness, and accuracy of the FRAP assay. For instance, it must be performed in an aqueous system, thus necessitating the use of a water-soluble reference antioxidant such as Trolox, ascorbic acid, or uric acid [23]. Since the oxidant in the “FRAP reagent” is not only Fe^3+^[TPTZ]_2_ but also other ferric species, evaluating the antioxidative potential of foods using this assay could be problematic because many metal chelators in food extracts are able to bind to Fe^3+^, forming complexes that have the capacity to react with antioxidants [18]. In addition, the assay is notorious for its inability to accurately measure the antioxidant activity of slow-reacting compounds such as polyphenols and for giving false-positive results for samples with redox potential values lower than the Fe^3+^/Fe^2+^ redox pair [15]. It has been noted that at approximately 0.70 V, there is little difference between the redox potential of the Fe^3+^ salt and that of the ABTS radical at 0.68 V, thus making differential pH (neutral for Trolox equivalent antioxidant capacity and acidic for FRAP) one of the only real differences between the two assays [18]. In the ferricyanide FRAP assay, it has been noted that the Prussian blue end product often precipitates to form a suspension, which contributes to staining the test cuvette and raising the potential for error in the assay [10].

### 2.4. Prooxidant Metal Chelation

Metal ions possess the capacity to induce lipid oxidation by means of the Fenton reaction as well as by breaking down lipid hydroperoxides into more reactive radicals [10]. Certain natural antioxidants including flavonoids such as quercetin, rutin, and (+)catechin have been shown to be powerful metal chelators [10,45]. Metal chelators function as antioxidants by scavenging ROS and also by decreasing the amount of available metals such as iron, thereby reducing the amount of hydroxyl radicals generated by Fenton reactions and limiting metal ion-induced lipid oxidation [10,46]. Since the antioxidant capacity of metal ion chelators is determined when a complex is formed between the antioxidant and the metal, which renders the metal ion unavailable to function as an initiator of lipid oxidation, metal chelation capacity is used as an indicator of antioxidant activity [10]. A common assay for investigating the capacity of biological samples to chelate metal ions involves the combination of FeCl_2_, ferrozine, and the test biological sample. Then, the absorbance readings at 562 nm is used as a measure of the metal ion-chelating capacity of the antioxidant sample [11,47]. Another adaptation of the assay uses ferrous sulfate in place of ferrozine and measures absorbance at 485 nm [10]. An important investigation of the metal ion-chelating property of various natural flavonoids found that flavonoids can act as both prooxidants and antioxidants depending on the nature and concentration of the flavonoids and metal ions [45].

### 2.5. Inhibition of Lipid Peroxidation including Lipoprotein Oxidation

Lipid oxidation is a major contributor to the impairment of food quality and industrial-scale economic losses [8]. In biological systems, lipid peroxidation greatly contributes to oxidative damage in cell membranes, lipoproteins, and other lipid-containing structures [48]. Iron-catalyzed one-electron reduction of lipid hydroperoxides can lead to free radical-mediated chain peroxidation and the perturbation of cell membrane structure and function and associated pathologies, while peroxyl radicals are known to play a vital role in the unsavory oxidation of lipids in food and biological systems [18,48]. In addition, when exposed to heat, light, enzymes, metalloproteins and metals, lipids are subject to oxidative processes, which could result in the development of rancidity and off-flavors and the consequent loss of essential organoleptic and nutritional qualities [8,10]. Lipid oxidation could be a result of autoxidation, photooxidation, thermal oxidation, or enzymatic oxidation [10]. The inhibition of linoleic acid oxidation by food extracts has been used to study lipid oxidation in vitro [8]. The assay typically involves the combination of an ethanolic linoleic acid solution with the antioxidant test sample and incubation at 60 °C in the dark for seven days [49]. Measurements are taken every 24 h for the entire duration of the incubation and typically entails aspirating and combining a specific amount of the previously incubated mixture with aqueous ethanol, ammonium thiocyanate, and FeCl_2_ and taking spectrophotometric measurements at 500 nm at room temperature [49]. Reductions in the absorbance value of samples when compared to blank reaction (containing no antioxidant molecules) are used as a measure of the inhibitory potency of the antioxidant compound against lipid peroxidation.

## 3. Cell-Based Antioxidant Assays

**Figure 3:**
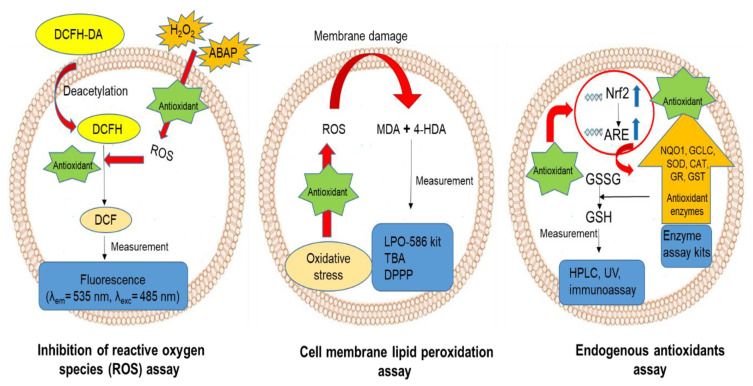
An illustration of some major intracellular antioxidant assays.

Cell-based techniques are considered the most popular antioxidant assay methods among in vitro, animal models, and human studies. Phytochemicals with in vitro antioxidant activity should have high bioavailability, distribution, and metabolism [50]. In vitro assays do not consider these biological parameters. Moreover, the best antioxidants activate intracellular responses such as the expression of enzymes (superoxide dismutase, catalase, glutathione peroxidase, etc.) and glutathione (GSH), which is a peptide that possesses radical scavenging activities. In addition, in vivo assays typically involve high costs and some ethical issues related to the excessive use of animals for routine research [51]. This underscores the importance of antioxidant evaluation in vitro at the cellular level. The major intracellular antioxidant assays discussed in this section are depicted in Figure 3.

### 3.1. Inhibition of Intracellular ROS Production

ROS, including different molecules with oxygen in their structure, are produced by cellular respiration in the mitochondria. These molecules can be free radicals such as nitric oxide, hydroxyl, peroxyl, and superoxide anion radicals, or non-radical species such as ozone, hydrogen peroxide, and singlet oxygen [52,53]. Oxidative stress is a condition characterized by imbalanced production and the removal of ROS by antioxidants. Extensive production of ROS and subsequent oxidative stress changes the redox status of cells and damages cellular proteins, lipids, and DNA, consequently changing their biological functions [54]. Diabetes, cardiovascular disease, aging, and neuronal disorders are common disease conditions and degenerative processes related to oxidative stress [53].

The cell-based antioxidant assay developed by Wolfe and Liu [55] has become a popular method for evaluating ROS inhibition by using an oxidation-sensitive fluorescence probe that is absorbable by cultured cells. Currently, 2′,7′-dichlorodihydrofluorescein diacetate (DCFH-DA) is commonly used as a probe. Non-fluorescent DCFH-DA is deacetylated inside the cells to form non-fluorescent DCFH, which is oxidized by H_2_O_2_ to form the fluorescent DCF. This cell-based method measures DCF formation in the presence and absence of antioxidants using fluorescence intensity (FI) measured at emission wavelength of 535 nm upon excitation at 485 nm [55]. Other than H_2_O_2_, 2,2-azobis (2-methylpropionamidine) hydrochloride (AAPH or ABAP) is also commonly used to trigger cellular oxidative stress. H_2_O_2_ and AAPH permeate the cells to produce peroxyl radical (ROO^•^), which causes tissue damage and cell death [56]. In this assay, there is an inverse correlation between FI and antioxidant activity of compounds. Antioxidants that scavenge peroxyl radicals induced by H_2_O_2_ prevent DCF formation and result in lower FI. Zebrafish larva, human colonic epithelial (HCT-116), hepatocarcinoma (HepG2), and colorectal adenocarcinoma (Caco-2) cells are common cell lines used to evaluate the ability of antioxidants to neutralize intracellular ROS [52,56,57]. Kellett et al. [58] compared the effectiveness of HepG2 and Caco-2 cell lines for measuring the biological antioxidant activity of food antioxidants and showed that the HepG2 cell line is not ideal for phenolic compounds. While flavan-3-ols such as (+)-catechin and (−)-epicatechin showed no antioxidant activity in HepG2 cells, they reduced FI by over 50% in Caco2 cells [58]. Moreover, the human erythrocyte is considered one of the best cell models for this assay. This is because they lack mitochondria and thus do not produce mitochondrial ROS that may influence the assay result [59].

Flavonoids have exhibited high intracellular antioxidant activity related to special structural properties. Having a 3-hydroxyl group, a 3′,4′-O-dihydroxyl group, and a 2,3-double bond in conjugation with 4-keto moiety potentiated high antioxidant activity [60]. Among flavonoids, quercetin displayed the highest antioxidant activity in the cell-based assay. Thus, quercetin equivalent is often used as a standard to express the cellular antioxidant activity. This method has been applied to evaluate the antioxidant activity of different phenolic extracts of fruits and vegetables, dietary fibers, and bioactive peptides. In a recent study, flavonoids with two OH groups exhibited higher antioxidant activity in vitro than in cell-based assays [61]. This result is expected, considering that cellular uptake of antioxidant compounds is a prerequisite for their effectiveness in cell-based assays. Low bioavailability is one of the main challenges associated with the use of flavonoids in pharmaceutical applications. However, catechin and its derivatives have higher bioavailability and intracellular antioxidant activity, which have been related to their lower molecular weight [53]. While caffeic and chlorogenic acids are strong antioxidants based on in vitro chemical assays, chlorogenic acid is poorly absorbable; this is related to the extracellular pH effect on chlorogenic acid absorption with no negative effect on caffeic acid [62].

Furthermore, the antioxidant effect of grape seed extract resulted in the restoration of mitochondrial membrane activity and decrease in ROS formation in the mitochondria and cells [63]. A synergistic intracellular inhibition of ROS production was reported for phenolic acid–carotenoid combinations with a higher concentration of phenolic acids. The phenolic acids increased carotenoid uptake by the cells, resulting in increased expression of membrane transporters and antioxidant activity. These findings show the importance of the antioxidant ratios and facilitated transport in obtaining the highest intracellular antioxidant activity [64]. In another study, peptides from split gill mushroom dose-dependently inhibited ROS production in HT-29 cancer cell line, and a peptide fraction with molecular weight of ≤ 0.65 kDa was found to be the most effective. Peptides with high lipid solubility are thought to be the best intracellular antioxidants. The lipophilic peptides can easily bind the cell membranes and penetrate inside the cells to inhibit lipid peroxidation and ROS formation. Therefore, high hydrophobic amino acid content in the peptide structure improves bioaccessibility and intracellular antioxidant activity [65]. *Saccharomyces cerevisiae* is another simple model used to evaluate the antioxidant activity of food compounds based on measurement of cell viability. This assay method evaluates the yeast cell tolerance against oxidative stress induced by ROS such as H_2_O_2_. Following incubation of yeast with antioxidants in yeast extract peptone dextrose, H_2_O_2_ is added, and cell numbers are estimated after 72 h by measuring colony-forming units of yeast [57]. Other than cell survival, point reverse mutation and antimutagenesis effects are two experiments used to show cellular damages induced by ROS and to evaluate the antioxidant activity of compounds in preventing these damages [59]. Table 1 shows different food-derived antioxidants evaluated by cell-based assays in recent studies conducted between 2015 and 2021.

### 3.2. Inhibition of Cell Membrane Lipid Peroxidation

Cell membranes are composed of lipids, which play significant roles in maintaining membrane fluidity and functionality. ROS can damage the membrane by oxidation of the lipids and causing membrane rearrangement, cell damage, and consequently inflammation, liver injury, atherosclerosis, and aging [74]. Malondialdehyde (MDA) and 4-hydroxyalkenals (4-HDA) are two cytotoxic end products of cell membrane lipid peroxidation [75]. MDA has a metabolic effect and is used as a significant marker of oxidative stress in organisms [52]. The LPO-586 kit is usually used to measure MDA and 4-HDA in cells [75]. This method is based on the interaction of N-methyl-2-phenylindole with MDA to produce a violet pigment with maximum absorption at 586 nm [76]. In another method, lipid peroxidation is measured using non-fluorescent diphenyl-1-pyrenylphosphine (DPPP), which forms DPPP oxide (fluorescent probe) in the presence of hydroperoxides [77]. DPPP oxide FI is measured at excitation and emission wavelengths of 380 and 340 nm, respectively. This method was used for the evaluation of the effect of taurine-rich *Paroctopus dofleini* extract on lipid peroxidation of zebrafish embryos induced by lipopolysaccharide. The extract effectively scavenged ROS and decreased lipid peroxidation in the cell membrane [78]. The antioxidant activity of curcumin evaluated by this method showed successful inhibition of lipid peroxidation induced by arachidonic acid [79]. Algae phlorotannins with high ROS scavenging potential also effectively inhibited lipid peroxidation in zebrafish embryo by reducing the formation of DPPP oxide [77]. Recently, DPPP was successfully applied in evaluating lipid and protein oxidation in fish muscles simultaneously [80]. Thiobarbituric acid (TBA) can also react with MDA to form a chromogen with maximum absorbance at 535 nm for assessing cellular lipid oxidation. For example, TBA was used to evaluate the effect of soy protein hydrolysates of different molecular weight ranges on cellular lipid peroxidation in Caco-2 cells. The ≤ 3 kDa hydrolysate was found to be the most effective, and this was likely due to its high hydrophobicity and effective cellular uptake [81]. Tyr and the hydroxylated aromatic ring of phenolic acids have the most important effect in controlling intracellular lipid peroxidation and protecting cell membrane integrity [82]. Red blood cells (erythrocytes) are a good choice of model for evaluating the antioxidant potential of food compounds, since they transport oxygen and have a high content of polyunsaturated fatty acids in the membrane. Flavonoids such as (−)-epicatechin gallate decrease oxidative hemolysis by spreading in the core of the bilayer membrane of red blood cells and inhibiting fluidity of the lipids. In this work, the presence of flavonoids decreased the penetration of alkly peroxide radicals, thus increasing membrane stability [53]. Hydrophobicity and amphipathicity of carotenoids and polyphenolic compounds are two main factors that affect their lipid peroxidation inhibition. These structural features facilitate the accessibility of the compounds to exhibit radical scavenging potential at the surface and bilayer of cell membranes. Based on these properties and polyphenolic contents, different degrees of cellular lipid oxidation inhibitory effects have been reported for extracts from fruits and vegetables [83].

### 3.3. Activation of the Endogenous Antioxidant System

Some antioxidant compounds show their effects by upregulating endogenous antioxidant enzymes and modulating gene expressions involved in oxidative stress. These enzymes include superoxide dismutase (SOD), catalase (CAT), glutathione S-transferase (GST), glutathione peroxide (GPx), γ-glutamyl cysteine synthetase (γ-GCS), and glutathione reductase (GR) [52]. SOD breaks down the superoxide radical to produce H_2_O_2_ or O_2_, CAT hydrolyzes H_2_O_2_ to yield H_2_O and O_2_, GSH eliminates H_2_O_2_ to become oxidized (GSSG), and GR reduces the GSSG back to GSH [52,56]. GPx and CAT are active enzymes in the second step of the antioxidant pathway where they scavenge intracellular H_2_O_2_; the two enzymes are considered to be more important than SOD [50]. On the other hand, vitamin C, GSH, α-tocopherol, β-carotene, and vitamin A are called the non-enzymatic antioxidant defense system of cells. These compounds are obtainable endogenously or through dietary sources to neutralize ROS. GSH is a tripeptide and non-protein thiol that plays an important role in the cellular defense system and redox state by the elimination of ROS and production of new antioxidants [84]. The ratio of reduced glutathione (GSH) to the oxidized form (GSSG) is an indicator of oxidative stress and can be measured by UV detection at 412 nm, HPLC, and fluorometric enzyme immunoassay [85]. GSH has an additional role in the detoxification of electrophilic xenobiotics including lipid peroxides through the activity of GST [50].

To evaluate the antioxidant enzyme activities (milliunits per mg protein) and total GSH in cells, colorimetric assay kits such as a total SOD assay kit with nitro blue tetrazolium, a CAT assay kit, a GR assay kit, and a total GSH assay kit are recommended. Using this approach, the antioxidant effect of eggshell membrane protein hydrolysate in Caco-2 cells was found to increase the cellular GSH level by increasing the mRNA expression and activity of γ-glutamylcysteine synthetase (γ-GCS). Increase in the activity of GPx, GST, and GR resulted in higher intracellular GSH level and an improved antioxidant defense system [76]. One of the main regulators of antioxidant enzyme expression is nuclear factor erythroid 2-related factor 2 (Nrf2). It acts by binding antioxidant response elements (ARE) to increase the expression of phase II antioxidant enzymes, such as heme oxygenase-1 (HO-1), NAD(P)H quinone dehydrogenase 1 (NQO1), and glutamate-cysteine ligase catalytic subunit (GCLC). Nuclear factor κB (NF-κB) is another important transcription factor associated with redox control. NF-κB binds to DNA after activation and increases pro-inflammatory/pro-oxidant gene expression [51]. Curcumin and blueberry extracts showed cellular antioxidant effects by inhibiting NF-κB activation [86,87]. Some food derived phytochemicals such as *p*-coumaric acid, caffeic acid, β-carotene, and lycopene derivatives improved the Nrf2 signaling pathway and resulted in higher expression of antioxidant enzymes. Combinations of phenolic acids and carotenes showed synergistic effects on intracellular Nrf2 expression in H9c2 cells [64]. Moreover, it was reported that increasing the expression of Nrf2 is the main mechanism of some bioactive peptides that showed intracellular antioxidant activity. Another important effect is the protection of the thiol-containing enzymes such as GR and thioredoxin [73]. The phenolic extract of cranberry beans has been shown to positively influence the antioxidant enzyme content of Caco-2 cells. Specifically, SOD, CAT, GPx, GR activities, and GSH production were stimulated by flavanols such as catechin, epicatechin, and proanthocyanidins. Catechin was shown to increase Nrf2 activity [66] and was more effective against endogenous radical than GSH [53]. Soy protein hydrolysate also increased CAT, GP, and GR enzyme activities, and GP was the most sensitive to peptide treatment. In addition, bioactive peptides with Tyr and Trp at the end of the sequence had high antioxidant activities by scavenging peroxyl radical and increasing the antioxidant activity of GSH through the addition of Tyr to the GSH N-terminal [72]. Overall, based on different studies and evaluation assay methods, it has been shown that bioactive peptides and hydrolysates show intracellular antioxidant activity via three mechanisms, including inhibition of ROS production, inhibition of lipid peroxidation, and activation of endogenous antioxidant enzymes [81].

## 4. Conclusions and Recommendations

The vital contributions of antioxidants to human health whether as a bulwark against ROS-induced oxidative stress and related chronic disease conditions or as protective agents against the oxidative deterioration of food products have continued to sustain interest in them by research scientists, healthcare professionals, dietitians, and even the lay public. Although comparatively little was known about antioxidants 30 years ago, recent advances in antioxidant research have uncovered crucial new information such as the strong antioxidant activity of polyphenols, effect of the position of phenolic –OH groups on antioxidant potency, quantitative structure active relationship data for the design of novel antioxidants, the role of food protein-derived bioactive peptides as antioxidants, as well as the importance of amino acid sequence on the efficacy of peptide-based antioxidant formulations, to mention a few [8,17]. Yet, in spite of the notable success of free radical chemistry and antioxidant research, it is widely accepted that the lack of effective, reliable, highly sensitive, and accurate antioxidant assay methods has continued to diminish opportunities for greater progress in this area of research [10,17,28]. Existing protocols vary in their oxidation initiator, ease of operation, substrate type, antioxidant mechanism, assay chemistry, and data analysis and reporting [10,17]. While it has been suggested that a universal and optimized assay protocol for determining antioxidant capacity is needful given the number of less than optimal assays in existence [14], it is a fact that no single method can realistically measure the total antioxidant activity of a biological sample. Apart from obvious technological challenges with designing such a universal or multifunctional assay given the differences and individual chemical properties of the various oxidants, the cost, complexity, and sophisticated tools that would be required to effectively use such an assay could prove prohibitive. It has been suggested that at least 2–3 different assays be used to measure antioxidant activity for greater reliability [14,23]. While combining the result of different assays could provide an approximation of the true total antioxidant capacity of a biological sample, such a practice is certain to be time-consuming, unwieldy, impractical, and challenging [9]. Thus, it is imperative to develop new, reliable, simple, more sensitive, highly specific, and selective antioxidant assays with clearly identifiable reaction mechanisms and fully tested reaction conditions [17,23]. Examples of efforts in this regard include the use of malondialdehyde/high-performance liquid chromatography (MA/HPLC) assay and malondialdehyde/gas chromatography (MA/GC) assay for measuring lipid peroxidation, using high-performance thin-layer chromatography coupled with DPPH bioautography (HPTLC-DPPH) for quantifying antioxidant activity in the medicinal plant *Alpinia officinarum,* and combining EPR spectroscopy with a free radical scavenging method such as DPPH for quantifying the various antioxidants in natural extracts such as pomegranate fruit juice [23,88,89].

Since the Prussian blue formed in the ferricyanide FRAP assay tends to form a suspension that could interfere with accurate assay readings, it has been suggested that the end product be stabilized against precipitation by adding NaC_12_H_25_SO_4_ as a surfactant and adjusting the reaction to pH 1.7 in order to maintain the Fe^3+^ redox activity without hydrolysis [90]. A recent adaptation of the FRAP assay, which employs electrochemical detection protocols and a chronoamperometric method for measuring antioxidant reducing power, has been recommended for evaluating the antioxidant activity of biological samples due to its low detection limit and increased accuracy, sensitivity, and reproducibility [91]. Therefore, while the search for a perfect antioxidant test remains elusive, current scientific developments suggest that a solution to this complex issue could be on the horizon.

## Figures and Tables

**Table 1:** Recent cell-based antioxidant assays for food-derived antioxidant components.

Ingredient	Cell-based Antioxidant Test	Cell Model	Antioxidant Effect	Reference
Lectin-free common bean (*Phaseolus vulgaris* L.)	CAA, haemolysis and antimutagenesis assay	Human erythrocytes, *Saccharomyces cerevisiae*	High dose-dependent antioxidant effect in yeast and human cells; attenuation of mutation induced by H_2_O_2_.	[59]
Turmeric leaf extract	CAA, lipid peroxidation and cell viability	Vero cells and zebrafish embryo model	Nuclear condensation, inhibition of ROS generation, cell death and lipid peroxidation.	[54]
Regular-darkening (RR) cranberry	Antioxidant enzyme activity	Caco-2 cells	Low permeability of flavanols; main influence was by surface adsorption and changes in cell signaling. Increase in level of SOD, CAT, GPx, GR, and GSH.	[66]
Fresh eating citrus fruits after in vitro digestion	CAA, cellular uptake assay	HepG2 cells	Digesta showed higher antioxidant activity than extract; strong correlation between naringenin and β-carotene absorption and antioxidant activity; detection of phenolic acids with hydroxybenzoic structure in cells showing permeability and no detection of hydroxycinnamic structure.	[67]
Flavonoid luteolin	GSH content, SOD activity, expression of antioxidant responsive-element (ARE) and Nrf2	Caco-2 cells	Increase in expression of ARE and Nrf2; higher level of GSH and SOD inside the cells.	[68]
*Bryophyllum pinnatum* leaf extracts	Lipid peroxidation, GSH content, SOD activity	Human red blood cells	Freeze-dried extract resulted in the lowest membrane destabilization, MDA formation, and highest GSH content and SOD activity.	[69]
Two bioactive peptides from brown rice hydrolysates	CAA and cell hemolysis	Human red blood cells	Dipeptide Leu-Tyr and tripeptide Tyr-Leu-Ala inhibited oxidation and decreased hemolysis rate.	[70]
Moroccan Zantaz honey rich in methyl syringate	CAA	Caco-2 cells	Methyl syringate containing more than 50% total polyphenols; antioxidant activity was mainly related to syringate and gallic acid contents.	[71]
Peptide fraction < 1 kDa from *Dendrobium aphyllum*	CAA in HepG2 cells, immune bioactivity in RAW 264.7 and cellular absorption in Caco2 cells	HepG2, Caco2, and RAW 264.7 cells	CAA in HepG2 cells was 63.46 µM quercetin equivalent/100 g of peptide; cytokine secretion increased in RAW 264.7 cells, uptake in Caco2 was 19.7–25.5%.	[72]
Milk-derived bioactive peptides	Lipid peroxidation, antioxidant enzyme and Nrf2 expression	Caco-2 cells	Main antioxidant mechanism involved increase in Keep1-Nrfs expression; inhibition of lipid peroxidation.	[73]
WL15 peptide from cysteine and glycine-rich protein 2	Lipid peroxidation, antioxidant enzyme activity and expression	Human erythrocyte and zebrafish embryos	Increase in SOD activity and gene expression of glutathione S-transferase, glutathione peroxidase, and γ-glutamyl cysteine synthetase. Decrease in caspase 3 expression and MDA production.	[52]
Corn gluten peptide fractions	CAA, antioxidant enzyme activity	HepG2 cells	High antioxidant effect was related to fraction < 1 and 1–3 kDa; increase in SOD, CAT, GR, and total GSH level.	[56]

CAA, cellular antioxidant assay; MDA, Malondialdehyde; SOD, superoxide dismutase; CAT, catalase; GPx, glutathione peroxide; GR, glutathione reductase; ROS, reactive oxygen species.

## Data Availability

Data are contained within the article.
